# Cytokines and Transgenic Matrix in Autoimmune Diseases: Similarities and Differences

**DOI:** 10.3390/biomedicines8120559

**Published:** 2020-12-01

**Authors:** Ludmiła Szewczak, Katarzyna Donskow-Łysoniewska

**Affiliations:** Laboratory of Parasitology, General Karol Kaczkowski Military Institute of Hygiene and Epidemiology, 01-163 Warsaw, Poland; katarzyna.d.lysoniewska@wihe.pl

**Keywords:** multiple sclerosis, systemic lupus erythematosus, T cells, cell signaling

## Abstract

Autoimmune diseases are increasingly recognized as disease entities in which dysregulated cytokines contribute to tissue-specific inflammation. In organ-specific and multiorgan autoimmune diseases, the cytokine profiles show some similarities. Despite these similarities, the cytokines have different roles in the pathogenesis of different diseases. Altered levels or action of cytokines can result from changes in cell signaling. This article describes alterations in the JAK-STAT, TGF-β and NF-κB signaling pathways, which are involved in the pathogenesis of multiple sclerosis and systemic lupus erythematosus. There is a special focus on T cells in preclinical models and in patients afflicted with these chronic inflammatory diseases.

## 1. Introduction

Organism homeostasis is determined by effective communication between different cell populations. This communication is possible due to secretion of special molecules, e.g., hormones, growth factors or cytokines. The action of these molecules is determined by binding to their receptors and signal transduction from receptors to nucleus where specific transcription factors modulate gene expression. Cytokines, including interleukins, interferons, chemokines, tumor necrosis factors or transforming growth factor-related family factors are crucial for communication between immune cells populations.

Multiple sclerosis (MS) and systemic lupus erythematosus (SLE) are autoimmune diseases in which MS is organ-specific and affects the central nervous system, whereas SLE can affect many organs and organ systems. The cytokine profiles in these two diseases show some similarities. The similarities include elevated serum levels of IL-6, IL-12, IL-17, IFNγ [1,2,3,4] and TNFα [5,6]. Despite these similarities, the cytokines have, to some extent, different roles in pathogenesis of these diseases. Other cytokines differ in their profiles, as in the case of IL-10, TGFβ and IL-4 [1,2,4]. The same cytokine can also have different roles in pathogenesis of each disease, e.g., interleukin-10 (IL-10) is thought to be one of the most important anti-inflammatory cytokines, but a high level of IL-10 in SLE exacerbates its course [7,8]. Altered levels or action of cytokines can result from changes in cell signaling.

For many interleukins, interferons and haematopoietic growth factors, signaling is based on Jak-STAT signal transduction. Cytokine binding leads to oligomerization of its receptor and activation of Janus Kinases (JAK) (JAK1-JAK3 and TYK2). Activated JAKs phosphorylate receptor domains that are docking sides for STAT (signal transducer and activator of transcription) proteins. There are seven STATs: STAT1-STAT4, STAT5A, STAT5B and STAT6. STAT transcription factors after phosphorylation form hetero or homodimers that translocate to the nucleus and regulate gene expression. The JAK-STAT pathway can be negatively regulated by eight SOCS (suppressors of cytokine signaling) proteins: SOCS1-SOCS7 and CIS [9,10]. Their mechanisms of action include direct inhibition of JAK, competition with substrates such as STATs, and targeting specific proteins to degradation through ubiquitination. SOCS proteins also regulate other signaling pathways, like those related to NF-κB [11]. Detected levels of proteins such as STAT do not always reflect an activation of signaling pathways, as only phosphorylated forms that migrates to the nucleus act as transcription factors. For example, STAT5B can migrate between the cytoplasm and the nucleus as an inactive monomer or as an active phosphorylated dimer [12], so its concentration does not have to reflect its activity.

Transforming growth factor β (TGF-β) and the TGF-β-related family of factors activate type I and II membrane receptors (TβRI and TβRII), that form homodimers. After TGF-β binding to the TβRII homodimer, the TβRI is recruited forming a hetero-oligomer. This receptor complex has both serine-threonine kinase activity and tyrosine kinase activity. Type II receptors phosphorylate type I receptors, activating them and allowing them to phosphorylate receptor-regulated Smads (R-Smads). Smad2 and Smad3 are R-Smads phosphorylated in response to TGF-β like proteins. Activation of R-Smads enables the formation of a complex consisting of two R-Smads and common-Smad (CoSmad)-Smad4. The complex then migrates into the nucleus where it regulates target gene transcription. Inhibitory Smads (I-Smads) antagonize the Smad signaling pathway and act as negative regulators of the pathway. There are two I-Smads: Smad6 and Smad7, and Smad7 antagonizes the Smad pathway related to TGF-β-like proteins signaling [13]. However, TGF-β is one of the most important anti-inflammatory cytokines, which promotes Th17 proinflammatory cells’ differentiation, together with IL-1β, IL-6, and IL-23 [14,15].

Cytokines belonging to the TNF family (TNFα, OX40, CD40, BAFF), IL-1 family (IL-1β, IL-18, IL-33) and IL-17 bind receptors, and receptor activation is associated with activation of NF-κB transcription factors and mitogen-activated protein (MAP) kinases such as p38 and JNK (c-Jun amino-terminal kinase). MAPK phosphorylates AP1 complex of transcription factors. This signaling can be negatively regulated e.g., by soluble receptor-like inhibitor, like IL-18BP [9,16]. There are two basic types of NF-κB pathway: canonical and noncanonical. The canonical pathway can be initiated by the TNF receptor (TNFR), IL-1 receptors (IL-1R) and Toll-like receptors (TLR), and the noncanonical pathway by e.g., B-cell activation factor (BAFFR), CD40, receptor activator for nuclear factor kappa (RANK) or TNFR2. Activation of the canonical pathway leads to activation of IKKβ kinase, being a component of the IKK complex, and leading to phosphorylation of IκBα. This leads to degradation of IκBα and release of bound NF-κB heterodimer that translocates to the nucleus [17]. The activation of IKKβ is the result of the action of many adapter proteins or kinases, including TRADD, RIP-1, TRAF2, FADD and precaspase-8 [18]. In the noncanonical pathway, activation of the receptor leads to activation of IKKα kinase by NIK (NF-κB inducing kinase) and IKKα phosphorylates p100 in a p100-RelB complex. Phosphorylation leads to ubiquitination, degradation of p100 and formation of p52-RelB NF-κB heterodimer that migrates to the nucleus [17,19].

This article describes alterations in three types of signaling pathways of cytokines: JAK-STAT, TGFβ and NF-κB signaling pathways, which are greatly involved in pathogenesis of multiple sclerosis and systemic lupus erythematosus, with special focus on T cells.

## 2. Multiple Sclerosis

Multiple sclerosis (MS) is a chronic, autoimmune, neurodegenerative disease affecting the central nervous system (CNS). Characteristic features of the disease are infiltration of leukocytes to CNS, demyelination and formation of demyelination plaques. This process causes a variety of neurological syndromes, symptoms and signs, including optical neuritis, cerebellar syndromes, motor deficits, like weakness or spasticity, sensory loss, chronic pain, sphincter dysfunction, sexual dysfunction, chronic fatigue and psychic disorders such as depression. Basically, there are four types of the disease basing on their course:

- Relapsing-remitting MS (RRMS)—the most common type, where episodes of neurological deficits (relapses) are separated by periods of remissions, where full or partial recovery occurs
- Secondary-progressive MS (SPMS)—sometimes developing over time in patients with RRMS, when the progression of neurological deficits becomes constant, without definite relapses and remissions
- Primary-progressive MS (PPMS)—the development of neurological deficits is constant from the beginning of the disease course
- Progressive-relapsing MS (PRMS)—the least common type, where clear relapses, periods of full, or not full recovery and constant progression of neurological deficits between relapses are present [20,21]

Immunopathology of the disease is multicellular. Autoreactive T cells infiltrates the CNS and contribute to the development of demyelinating lesions. The most important lymphocyte types in MS pathogenesis are Th17 and Th1 CD4+ T cells, producing IFNγ and IL-17A. While effector CD4+ T cells are considered to initiate the lesions, CD8+ T cells are proposed to amplify them, directly damaging axons. MS is also associated with Treg cell dysfunction and expansion of autoreactive B cells [22,23].

### 2.1. JAK-STAT Pathways in Multiple Sclerosis

In multiple sclerosis, harmful effector T cells are resistant to suppression by regulatory T cells. Resistance of effector cells is present in active, but not in inactive disease. It is caused by overexpression of the IL-6 receptor α (IL-6Rα) on the CD4+ T cell surface. That increases phosphorylation of pSTAT3 in response to IL-6. Blockade of STAT3 phosphorylation can reverse the resistance of the effector T cells [24]. STAT3 is crucial for Th17 cell co-differentiation and IL-17 production. CD4 Stat−/− mice do not develop EAE (a mouse model of multiple sclerosis) or EAU (autoimmune experimental uveoretinitis), have impaired Th17 cell differentiation and an increase in T cells expressing Foxp3, IL-10, IL-4 and IFNγ [25].

Some alterations in mRNA expression of STAT proteins have been observed in multiple sclerosis. In peripheral blood cells, there is downregulation of STAT5a and upregulation of STAT6, correlating with EDSS score [26]. Another study showed upregulation of STAT1 and downregulation of STAT2 mRNA expression in the blood of MS patients, with no significant difference in STAT3 expression between MS patients and healthy controls [27].

Granulocyte-macrophage colony-stimulating factor (GM-CSF) is one of the key activators of T cells pathogenicity [28] and autoimmune neuroinflammation. However, at the transcriptional level, the mechanism of GM-CSF generation is not clear. One way of GM-CSF stimulation in experimental autoimmune encephalomyelitis (EAE) is the IL-23/RORγt pathway. IL-12 and IFNγ are negative regulators of this pathway of GM-CSF generation [29]. GM-CSF production by Th17 cells is stimulated by IL-23 and further GM-CSF production gives a positive feedback loop for APC (antigen presenting cells) to produce IL-23 [30]. Specific, pathogenic GM-CSF producing Th cells in the central nervous system require IL-23R and IL-1R signaling, but not IL-6R, unlike classic Th17 cells. However their pathogenic role probably is not limited to GM-CSF production [31]. Another study identifies IL-1β as the most important factor stimulating GM-CSF. The proposed mechanism is RORγT—independent and includes ubiquitination of IRAK1 (IL-1 associated kinase 1) and NF-κB activation [32]. STAT5 also seems to be an important factor for generation of neuroimmunopathogenic T CD4+ cells in EAE. STAT5 activated with IL-7 promotes generation of GM-CSF/IL-3 producing CD4+ T cells that are a distinct from Th1 and Th17 lymphocytes. Loss of STAT5 function results in a reduction of EAE development [33]. However, some studies indicate that production of GM-CSF by Th cells is induced via the IL-12/T-bet/Th1 cell axis but constrained by the IL-23/RORγT/Th17 axis. Also in naïve and memory Th cells, IL-2-induced STAT5-stimulated GM-CSF production, whereas STAT3 signaling had an opposite effect [34]. STAT4 stimulates GM-CSF production by Th1 and Th17 cells in EAE mice [35]. IL-12 induced Th1 cells producing IFNγ and GM-CSF induced severe EAE, whereas IL-23-induced Th17 cells induced EAE with a milder course, independent of the IL-12 pathway [36].

One of the most important pathways in pathogenesis of multiple sclerosis is the IFNγ/STAT1/T-bet pathway of Th1 cells. There is upregulation of T-bet, pSTAT1and pSTAT3 in both CD4+ and CD8+ T cells and also in monocytes of patients with RRMS during relapse compared to those in remission and healthy controls. Higher levels of these factors correlate with the size of MRI imaged brain and spinal cord lesions There is also a strong correlation between pSTAT1 level and T-bet expression, and elevated production of IFNγ by PBMC. pSTAT3 upregulation was associated with higher production of IL-10, but not IL-6 [37]. There is also a lower percentage of circulating CD4+ CD25+Foxp3+ T cells, with lower capacity to suppress proliferation of autologous CD4+CD25+ T cells and a higher percentage of CD4+T-bet+ T cells with higher T-bet expression in RRMS patients, both in relapse and remission, than in healthy controls [38]. In early MS, there is increased abundance of CCR7+ and IL-6+ T cells and decreased abundance of NFAT1^hi^T-bet^hi^CD4+ T cells [39]. Another study showed that Th17 cells of patients with active MS have increased expression of mRNA and surface protein IFN-γR2 and corresponding JAK2, compared to nonactive disease and healthy control group [40]. These data show dysregulation of IFNγ signaling in MS at many levels and in distinct T cell populations.

A slight decrease in STAT phosphorylation is observed in PBMC of RRMS and SPMS compared to healthy controls. STAT1 phosphorylation levels are decreased in CD4+ T cells and monocytes and STAT6 phosphorylation levels are decreased in CD8+ T cells. However, dramatic increase of STAT and p38MAPK phosphorylation is observed after in vitro stimulation with IFNα of all PBMC populations. This increased phosphorylation is also present in MS patients, except for p38MAPK phosphorylation in CD8+ T cells. Similarly, all splenocytes derived from MOG—induced EAE mice had increased phosphorylation of STAT1 after in vitro stimulation, and also an increase of pSTAT4 and pSTAT5 in CD4+ T cells [41].

A less well-known subpopulation of T lymphocytes—Th22, is involved in pathogenesis of chronic inflammatory diseases [42]. In MS, there is an increased number of autoantigen-specific Th22 cells in peripheral blood and cerebrospinal fluid, especially before the active phase of the disease. These cells have high expression of CCR6 and T-bet. The Th22 cells are also insensitive to IFNβ treatment [43].

SOCS1 and SOCS3 are important regulators of Th1 and Th2 differentiation. SOCS1 is an inhibitor of interferon, IL-12/23, IL-4/13 and also IL-2 family cytokines and is an important factor in CD8+ T cell differentiation. SOCS3 is an inhibitor of IL-6 family cytokines, G-CSF and leptin. It inhibits the differentiation of Th cells into Th1 and Th17 cells. SOCS1 level is higher in Th1 cells than in Th2 cells, inhibiting the IL-4/STAT6 axis in these cells, whereas SOCS3 level is higher in Th2 cells, inhibiting the IL-12/STAT4 axis [44]. Expression of SOCS proteins in MS is also altered. In RRMS patients’ blood SOCS1 and SOCS5 are downregulated, but expression is not correlated with the EDSS disability score [45]. SOCS3 is upregulated in peripheral blood leukocytes and there is negative correlation between level of SOCS1 and SOCS3 in multiple sclerosis patients [46]. Other data show that SOCS1 mRNA [47] is highly overexpressed in PBMC of RRMS patients taking an interferon-beta-based drug compared to healthy controls. SOCS1 is especially important in CD8+ T cell proliferation control. This protein diminishes CD8+ activation and proliferation upon IL-15 and self—ligands stimulation. SOCS1 deficiency can lead to uncontrolled CD8+ T cell proliferation, hyperactivation and autoimmunity [48,49]. Th1 and Th2 cells from SOCS1−/− and +/− mice have enhanced functions and produce significantly more IFNγ and IL-4 respectively [50].

T cells of MS patients reveal impairment of the IL-10 signaling pathway. Tr1 lymphocytes induced in vitro from CD4+ MS patients produce less IL-10 than those from healthy donors and have higher expression of SOCS3 mRNA. After in vitro Il-10 stimulation, cells from patients with MS reveals higher expression of STAT1, STAT3 and IL-10RA mRNA and higher level of phosphorylated STAT3 [51]. Th17 cells of MS patients have higher expression of IL-10 than healthy controls and IL-10 expression is higher in clinically stable than in active patients [52].

There are several alleles of C-type lectin like domain family 16, member A (CLEC16A) connected with a higher risk of multiple sclerosis. Thymic samples with at least one autoimmune-risk allele of CLEC16A SNPs (rs12708716, rs6498169 and rs7206912) had lower expression of SOCS1 and DEXI (dexamethasone-induced protein) genes compared to noncarrier samples. There was also correlation between the expression level of CLEC16A and that of SOCS1 and DEXI in thymic samples [53]. There is higher expression of SOCS1 and CLEC16A in whole blood-derived CD4+ T cells in samples homozygous for the risk allele of CLEC16A rs12927355. There is also a high correlation between gene expression in peripheral T cells of CIITA, DEXI, CLEC16A and SOCS1 [54].

Expression of FOXP3, EBI3 and GATA3 in lymphocytes of RRMS patients decreased, with an increased RORC/FOXP3 gene expression ratio [55]. Overexpression of RORC in γδ T cells and iNKT cells of RRMS patients in relapse was also detected [56].

Gene candidates associated with higher risk of MS include regulators of NF-κB signaling and regulators of CD4+ Th1 and Th17 cells [57]. There is also an association between some MS-risk alleles and increased mRNA levels of JAK1 and STAT4 in CD8+ T cells, as well as decreased levels of TYK2 mRNA in CD4+ T cells. However, no significant differences were detected in gene expression levels of these proteins between lymphocytes of RRMS patients and healthy controls [58]. These molecules belong to the IL-6, IL-12 and IL-23 signaling pathway. Higher production of proinflammatory TNFα and susceptibility to multiple sclerosis is also associated with some genetic variants in the NF-κB signaling cascade, such as variants proximal to NFκB1 and in an intron of TNFRSF1A (TNFR1), which leads to higher NF-κB signaling after stimulation with TNFα [59].

The 77 C-G mutation in the CD45 gene is more common in T cells from patients with multiple sclerosis than those from healthy individuals. The mutation leads to higher susceptibility to multiple sclerosis. In vitro-generated T cell lines with the 77 C-G mutation in the CD45 gene show enhanced TCR signaling leading to higher IL-2 production and proliferation [60,61].

Some MS patients have increased expression of interferon-β-inducible genes in peripheral blood mononuclear cells. These patients have decreased CD4+ T cells reactivity to the autoantigen myelin basic protein ex vivo, similar to patients treated with IFNβ. This process can be mediated by monocyte-derived IL-10 [62]

### 2.2. TGFβ Signaling Pathway in Multiple Sclerosis

In MS, TGFβ signaling pathway alterations were also detected. Expression of SMAD7 mRNAs in CD4+ peripheral T cells was decreased during both relapse and remission in MS patients. SMAD2, SMAD3 and SMAD4 genes expression was unchanged compared to healthy controls. SMAD7 negatively regulates TGFβ signaling pathway, thus its decreased expression can lead to increased expression of TGFβ and promotion of Th17 polarization [63]. CD4+ T cells of MS patients showed higher expression of TGF-βRII and SMAD4 and decrease of SMAD7 expression, compared to healthy controls, whereas SMAD3 expression was unchanged [64]. In another study, a set of micro RNAs differentially expressed in MS patients CD4+ T cells targeting TGFβ signaling, was typed. The differentially expressed miRNAs reduced TGFβ signaling, leading to a reduction in naïve CD4+ T cell capacity to differentiate into Treg cells [65].

### 2.3. TNFα and IL-17 Pathways in Multiple Sclerosis

TNFα signal transduction in lymphocytes is altered in multiple sclerosis. Fas-associated death domain protein (FADD) gene expression was upregulated in leukocytes from RRMS patients compared to SPMS, PPMS and healthy controls [66]. TRAF2 (TNF receptor-associated factor 2) gene expression was increased in leukocytes of RRMS patients compared to other MS forms and healthy controls. In addition, RIP (receptor-interacting protein) expression was elevated in peripheral blood leukocytes of RRMS, SPMS and PPMS patients compared to healthy controls [67].

Th17 T cells play an important role in the pathogenesis of MS. In active MS lesions there were increased number of Th17+ cells, but also astrocytes and oligodendrocytes. Foxp3+ cells were not detected in active lesions [68].

The role of Hectd3—E3 ubiquitin ligase is proposed in the process of Th17 cell generation. Hectd3 polyubiquitinylates STAT3 and Malt1 led to NF-κB activation and RORγt production. In this mechanism, RORγt+IL-17Ahi effector CD4+ T cells are generated in EAE [69]. The p38 pathway is also involved in generation of Th17 cells from CD4+CD27+CD45RA+ naive T cells and release of IL-17 from Th17 and central memory lymphocytes. In RRMS patients cells the responsiveness of p38 cascade is higher [70].

In RRMS patients, the levels of IL-17 are elevated even during remission, and connected with IL-6R signaling. In vitro treatment of RRMS patients’ T cells with antiIL-6R monoclonal antibody caused a decrease in IL-17 production and an increase in IL-10 production by activated CD4+ T cells but didn’t affect CD8+ T cells. The IL-6R blockade increased T cell response to hydrocortisone, causing cell proliferation and inhibition of release of IL-17 [71]. However, neutralizing Th17 itself seemed to be an unsatisfactory method for blocking RRMS in the animal model of EAE and MS patients [72,73] Th17 plays a crucial role in pathogenesis and modulating the immune response in MS.

### 2.4. Impact of Drugs on T Cell Signaling in Multiple Sclerosis—Examples

Interferon beta (IFNβ) is a common but sometimes ineffective treatment of multiple sclerosis. Its action depends on the immune cell subtype. IFNβ strongly influences CD8+ T cells action, leading to decrease of IFNAR2, Tyk2, IRF9 and Jak1 gene expression and increased expression of the MxA gene [74].

Several monoclonal antibodies are used in MS treatment. Natalizumab is a humanized monoclonal antibody against α4-integrin (CD49d). During natalizumab treatment of MS patients, there was an increase in the expression of TBX21, RORC, IFNγ, and IL-17A, and a decrease in the expression of FOXP3 in CD49d+ memory CD4 T cells. The amounts of IFNγ and IL-17A secreted by CD49d+ memory CD4 T cells also increased. The CD49d+ Tregs population reduction was greater than that of Th1 and Th17 cells. These results provide an explanation for disease activity during the treatment [75].

The CD46-costimulation pathway in Tr1 cells is impaired in multiple sclerosis, leading to decrease in IL-10 production [76]. Calcitriol can modulate the CD46 pathway that is impaired in MS patients. Administration of calcitriol affects the phenotype of CD46-costimulated CD4+ T cells, increasing expression of CD28, CTLA4 and Foxp3 and decreasing CD25 expression, despite the fact that, normally, CD46 pathway activation induces CD25 expression. Change of phenotype results in an increase of the IL-10/IFNγ production ratio in Tr1 cells [77].

Fingolimod (FTY720) is an immunomodulating prodrug used in MS treatment. After phosphorylation p-FTY720 acts as an analog of sphingolipid sphingosine-1-phosphate (S1P), involved in immunomodulatory processes [78]. FTY720 inhibits TCR-dependent and TCR-independent activation of primary T cells. FTY720 doesn’t affect proximal TCR signaling but inhibits distal TCR signaling, reducing IL-2 and IFNγ release and CD25 expression. This drug also induces aberrant NFAT1, AP1 and NF-κB activation [79]. In CD8+ T cells and splenocytes derived from Sphingosine kinase 2 (SphK2)-deficient mice, unphosphorylated FTY720 inhibits IL-33/IL12-stimulated IFNγ release. This process is mediated via the SET/protein phosphatase 2A (PP2A) pathway [80].

Although the impact of immunomodulatory drugs on cytokine levels in MS is well researched [81], there is poor evidence concerning the impact of other immunomodulating drugs used in multiples sclerosis treatment on lymphocyte signaling pathways. However, some research on these drugs was carried on other in vitro models. Dimethyl fumarate (DMF) activated SOCS3, thus leading to repression of JAK1 and STAT3 phosphorylation in a hepatocellular carcinoma in vitro model [82]. There is also evidence on limiting the IL-17 and RORγT expression by DMF and inhibiting IL-2-STAT5 signaling in multiple sclerosis, leading to reduced frequency of Tc17 cells [83]. Another drug, teriflunomide, inhibited STAT6 phosphorylation in a porcine jejunal epithelial cell line IPEC-J2 [84].

## 3. Systemic Lupus Erythematosus

Systemic lupus erythematosus (SLE) is an autoimmune disease of connective tissue causing a broad range of symptoms and affecting many organs including skin, joints, kidneys (lupus nephritis), circulatory and respiratory systems, bone marrow and, in rare cases, the nervous system. The set of autoantibodies is produced by B cells, and immunological complexes damage affected organs. The plasma cell number is elevated and there is reduction in the number of naïve B cells. T cell response to antigens is altered due to changed composition of CD3, where the CD3-ζ chain is replaced by the FcR-γ chain. There is also a reduction of IL-2 production and elevated levels of IL-17 [85].

### 3.1. Jak-STAT Signaling in SLE

In the MRLlpr murine model of SLE, many alterations in cytokine signal transduction were noticed during progression of the disease, including impairment of STAT1 signaling in response to IFNα, IFNγ, IL-6, and IL-21, STAT3 signaling in response to IL-6, STAT5 responses to IL-15 and STAT6 responses to IL-4. There was also an increased response to IL-10 by T cells and increased expression of SOCS1, correlating with negative regulation of the STAT1 response to proinflammatory cytokines [86]. These observations were confirmed in another study where peripheral-blood mononuclear cells of lupus patients were investigated. T cells of lupus patients showed poor response of STAT1, STAT3 and STAT5 to IFNα [87]. The levels of serum IL-10, IL-6 and TNFα were increased in SLE such as intracellular expression of IL-10 in CD4+ and CD8+ T lymphocytes [88]. These data show that T cells in SLE undergo complex changes in responses to cytokines, thus leading to alterations of their functions.

The IFNγ signaling pathway seems to be important in pathogenesis of SLE. In both lymphocytes and monocytes of SLE patients there is increased gene expression of STAT1. The level of STAT1 expression correlates with disease activity and expression of CD95 and HLA-DR. In monocytes of SLE patients, expression of the IFN-inducible genes IP-10 and Mig is increased [89]. STAT1 levels in CD4+ T cells from lupus patients are higher than from rheumatoid arthritis patients and healthy subjects and positively correlate with disease activity, with highest levels in CD45RA-FOXP3hi-activated Tregs (aTregs). This subset also had the highest STAT1 phosphorylation response, and proliferation marker Ki-67 significantly decreased after IFNγ stimulation suggesting, that increased STAT1 signaling impaired aTregs function [90]. The CD8+ T cells of SLE patients have 188 hypomethylated CpG sites compared to healthy controls, especially in HLA-DRB1 and genes associated with type-I interferon response, including STAT1. This changed CD8+ T cell response to IFNα: upregulation of HLA-DRB1 in SLE patients CD8+ T cells and STAT1 in both SLE and control CD8+ T cells [91]. Changed phenotype of CD8+ T cells in lupus, and altered response to type I IFN, may indicate dysregulated STAT1 signaling in these cells. The transcriptomic research of Li et al. typed JAK2, STAT1, and STAT2 as crucial genes in pathogenesis of SLE [92]. JAK2 mRNA is overexpressed in PBMC of SLE patients [93], indicating that IFNγ signaling can be dysregulated at different levels.

The STAT4-IL-12 axis is related to Th1 response and is another important pathway in the pathogenesis of SLE [94]. In lupus, serum levels of IL-12 are increased, and increased production of IL-12 has an impact on lupus nephritis development [95]. Another study showed overexpression of IL-12 mRNA in PBMC from SLE patients. In this study IL-12 treatment of PBMC from SLE patients caused increased phosphorylation of STAT4 and STAT3 and no characteristic changes in PBMC of healthy controls [96]. In a Jordanian population of SLE patients, expression of IRF5, TLR-7, MECP2, STAT4, and TNFSF4 genes was investigated. All the genes were overexpressed in PBMC compared to healthy controls. STAT4 and TNFSF4 gene expression level, and IL-10 serum level, correlated with cardiovascular damage. In addition, TNFSF4 gene expression level correlated with pulmonary and musculoskeletal damage [97]. There is also a lot of evidence that some STAT4 polymorphisms can contribute to SLE development. A variety of studies found alleles of the STAT4 gene that affected the levels of STAT4 [98,99]. In PHA/IL-2-activated CD8+ T cells of SLE patients carrying the risk allele of STAT4 rs7574865[T], higher levels of STAT4 and increased pSTAT4 levels in response to IL-12 and IFN-α were detected. There was also increased production of IL-12-induced IFN-γ by CD4+ and CD8+ T cells [100]. Interestingly, the same risk allele exerted an opposite effect in T cells of healthy individuals [101]. Studies carried out on mice revealed distinct roles for STAT4 and STAT6 in the pathogenesis of lupus, indicating that STAT4 can be necessary in the process of autoantibody production, whereas STAT6 may play a role in the development of glomerulosclerosis [102,103]

STAT3 signaling is involved in pathogenesis of lupus erythematosus in several ways. First, there was dysregulation of the IL-17/STAT3 axis in lupus resulting in elevated IL-17 and higher numbers of Th17 cells. CD4+ T cells from lupus patients have higher expression of IL-17 and SOCS3 than healthy controls, with positive correlation between IL-17 levels and SOCS3 expression. There were also higher intensities of pSTAT3/β-actin and STAT3/β-actin in lupus with a positive correlation of these intensities with IL-17 levels. The level of IL-17 was also higher in lupus nephritis than in lupus without kidney involvement [104]. T cell-specific silencing of STAT3 in lupus-prone mice resulted in amelioration of lupus nephritis, probably due to impairment of T cell stimulation of B cells to produce dsDNA autoantibodies [105]. Moreover, miR-410 suppresses activity of STAT3, resulting in decreased production of IL-10 by T cells. Expression of miR-410 in T cells, especially in CD4+ T cells of SLE patients, is decreased and IL-10 levels increased, compared to healthy individuals. Overexpression of miR-410 resulted in decreased IL-10 expression. Silencing of STAT3 by miR-410 resulted in decreased IL-10 expression in CD3+ T cells [106]. STAT3 also inducedSTAT3-dependent Th17-specific regulatory T cells (Treg17) [107]. Foxp3 (Cre) × Stat3 (fl/fl) mice with pristine-induced lupus revealed enhanced peritoneal inflammation caused by lack of Treg17 and increased the percentage of Th17. These mice developed pulmonary vasculitis with increased mortality and lupus nephritis. Moreover, the expression of Th17-trafficking CCR6 was reduced on Tregs resulting in reduced infiltration of these cells in kidneys [108].

There is also a unique population of IL-17 producing RORγt+Foxp3+ T cells, biTregs, that have both regulatory and proinflammatory functions. In a mouse model of pristane-induced lupus, expansion of these cells was simultaneous with autoimmunity development and tissue injury. BiTregs also suppressed the anti-inflammatory action of Th2 cells in a RORγt—dependent manner [109].

There is dysregulation of STAT5 phosphorylation in CD4+ T cells from SLE patients. In conventional CD4+ T cells (T con) and CD4+ Foxp3lo cells, there were higher pSTAT5 levels, and in CD45RA+Foxp3hi-activated Tregs (aTregs), there were lower pSTAT5 levels than in RA patients or healthy controls. Bcl2 expression in Tcon was elevated due to IL-7-dependent STAT5 phosphorylation and aTreg cells Bcl2 expression was reduced. Enhanced expression of Bcl2, an antiapoptotic transcription factor, can promote proliferation of autoreactive CD4+ T cells in SLE. The percentage of proliferation marker Ki-67-expressing CD4+ T cells is also elevated in SLE. Moreover, patients with elevated Tcon pSTAT5/aTreg pSTAT5 ratio had more severe relapse course [110]. Another study showed that naïve CD4+ T cells from lupus patients had impaired production of IL-2. The proliferative response to IL-2 was reduced in these cells compared to cells from healthy subjects, due to low JAK3/STAT5 phosphorylation [111].

There are some alterations in SOCS proteins expression in SLE. Studies showed distinct alterations in SOCS1 expression. Decreased expression of SOCS1 mRNA in PBMC of patients with SLE was detected, compared to healthy individuals [112], whereas Chan et al. showed overexpression of SOCS1 mRNA in PBMC of SLE patients with higher levels in active disease [113]. Interestingly, SOCS-1−/− mice developed fatal disease with a course similar to SLE, presumably due to hyperactivation of CD4+ T cells and impaired function of CD4+CD25+ regulatory T cells [49]. In another study, the role of CIS in pathogenesis of SLE was proposed. In this research, there was overexpression of CIS mRNA in PBMC in the course of active disease, without changes in SOCS1, SOCS2 and SOCS3 mRNA levels, compared to healthy individuals and patients with inactive disease [114]. Moreover, there is evidence on alterations in epigenetic regulation of the set of genes in CD4+ T cells of patients with SLE. In these genes, including SOCS1 and IL-15RA there was increased hydroxymethylation of promoters and overexpression of these genes [115].

In CD4+ Tregs of SLE patients IL-21 resulted in increased activity of mTORC1 and mTORC2, leading to a decrease in autophagy, GATA-3 and CTLA-4 expression and impaired Treg suppressive functions. Four-week therapy with rapamycin restored Treg functions and normal expression of GATA-3, CTLA-4 and autophagy by blocking mTORC1 and mTORC2 [116].

### 3.2. TGF-β Signaling in SLE

In SLE, there are also alterations in TGF-β signaling, although little is known about TGFβ signaling in T cells during this disease. Transcription of TGF-β1 in response to stimulation of CD3+ T cells with TGF-β1 was impaired in active SLE patients. This impairment did not depend on alterations in TGF-βRII expression or Smad2/3 phosphorylation and did not correlate with enhanced IL-15 expression. Nevertheless, it was associated with IL-22 overexpression [117]. Another study demonstrated decrease in retinoid acid-induced expansion of TGF-β-induced Tregs. In CD4+ T cells from SLE patients, expression of homeotic gene Pdx-1 isoform—Pdx-1-d was increased and significantly reduced the expansion of CD25-Tregs, a population without suppressive activity. Increased expression of Pdx-1-d also prevented the reduction of activated CD4+CD25+FOXP3- T cells by TGF-β and retinoic acid [118].

The study of Lewis et al. showed some new targets of autoantibodies in SLE, including autoantigens connected with Smad2, Smad5 and other proteins linked to TGFβ signaling, MyD88 and proteins involved in TLR signaling, apoptosis and NF-κB signaling and T cell development [119]. These data indicate a possible reason for dysregulation of T cell signaling in lupus.

### 3.3. IL-1, TNFα and IL-17 Signaling in SLE

PBMC cDNA microarray analysis revealed a set of genes in which alterations of expression may play a role in SLE pathogenesis: TNFα, IL-1 family cytokines and their receptors [120].

One of the identified risk genes for SLE is IRAK1 (interleukin-1 receptor associated kinase-1) [121]. The study of Zhou et al. showed, that CD4+ T cells of SLE patients had higher expression of IRAK1, compared to healthy subjects, and increased expression was positively correlated with disease activity. Phosphorylation of IRAK1 in SLE was also enhanced after IL-1β in vitro treatment. IRAK1 mRNA expression was also positively associated with Th17/IL-17A in SLE patients. Repression of IRAK1 in CD4+ T cells differentiating into Th17 resulted in decreased IL-17A production and inhibited expression of genes typical to Th17 cells: RORC, IL-23 receptor and IL-17A [122].

The signaling pathway of TNFα was dysregulated, including decreased mRNA expression of TNFα adapter proteins in PBMC, such as TNF receptor-associated death domain (TRADD) protein, Fas-associated death domain (FADD) protein, receptor-interacting protein 1 (RIP-1), and TNF receptor-associated factor-2 (TRAF-2). In addition, there was overexpression of Caspase-3 while there was no significant changes in IL-1β mRNA expression, compared to healthy controls [123].

Another mechanism underlying inflammation and lymphocyte hyperactivity in SLE may be a result of alterations in JNK and MAPK signaling pathways. In CD4+ T lymphocytes, CD8+ T lymphocytes and B lymphocytes of SLE patients, increased expression of phospho-p38 MAPK was detected. The phosphor-JNK expression was increased in CD8+ T cells and B cells after activation with IL-18 [124].

### 3.4. Impact of Drugs on T Cell Signaling in Systemic Lupus Erythematosus—Examples

Hydroxychloroquine (HCQ) is an antimalarial drug used in treatment of rheumatic diseases, including SLE. The drug suppressed CD154 expression in in vitro-activated T cells of lupus patients. The effect was caused by inhibition of NFAT signaling, whereas STAT5-dependent CD154 expression was unaffected [125].

The immunosuppressive drug mycophenolate mofetil (MMF) inhibited STAT3 phosphorylation and reduces STAT3 phosphorylation in PBMC in vitro after IL-6 stimulation. Some lymphocyte populations, including Th17-type cells (CCR6+CD161+) and Treg-type cells (CD25hiCD127−) were reduced after MMF treatment [126].

Glucocorticoids are widely used in treatment of autoimmune diseases, including SLE. Prado et al. revealed an increase in the Th17/Th1 ratio in SLE patients treated with glucocorticoid, compared to healthy controls and patients untreated with glucocorticoids. In PBMC of treated SLE patients, overexpression of STAT3 and IL-6R was detected, with a positive correlation between STAT3 level and the Th17/Th1 ratio. This data may indicate that not only a disease, but also its treatment, can contribute to Th17 response imbalance [127].

## 4. Conclusions

**Figure 1:**
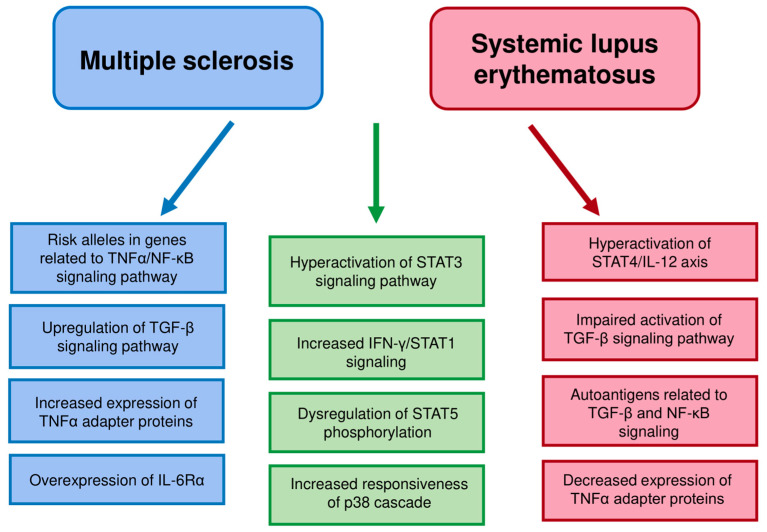
The main similarities and differences in T cells pathway dysregulation between MS and SLE. Similarities are marked in green. The MS dysregulation features distinct from SLE are marked in blue and SLE dysregulation features distinct from MS in red.

Multiple sclerosis and systemic lupus erythematosus are distinct autoimmune disease, but show certain similarities, including increased STAT3 expression and activation or increased SOCS3 expression. Some kinds of dysregulation in cell functioning can be common in different autoimmune diseases. Signaling pathways can differ between distinct T cell populations, as in the case of STAT1 and STAT5. Some cell signaling pathways differ between the diseases, e.g., TNFα signaling pathways (Table 1 and Figure 1). The alterations in cell signaling can be different in the two diseases despite similar changes in cytokine production.

## Figures and Tables

**Table 1:** Comparison of quantitative alterations in proteins of JAK-STAT, TGF-β and TNFα signaling pathways in multiple sclerosis and systemic lupus erythematosus. Arrows indicate a decrease/an increase and “?”—no data. A “p” denotes a phosphorylated residue.

	Multiple Sclerosis	Systemic Lupus Erythematosus
JAK1	↑ JAK1 mRNA in CD 8+ T cells [58]	?
JAK2	↑ JAK2 and JAK2 mRNA in Th17 cells [40]	↑ JAK2 mRNA in PBMC [93]
TYK2	↓ TYK2 mRNA in CD4+ T cells [58]	?
STAT1	↑pSTAT1 in PBMC [37] ↓pSTAT1 in CD4+ T cells [41] ↑STAT1 mRNA in blood [27]	↑STAT1 mRNA in T cells [89] ↑STAT1 in CD4+ T cells [90]
STAT2	↓STAT2 mRNA in blood [27]	?
STAT3	↑pSTAT3 in CD4+ T cells [24] ↑pSTAT3 in PBMC [37] No changes in STAT3 mRNA expression in blood [27]	↑STAT3 and pSTAT3 [104]
STAT4	↑ STAT4 mRNA in CD8+ T cells [58]	↑ STAT4 mRNA in PBMC [97]
STAT5	↓ STAT5a mRNA in PBMC [26]	↑pSTAT5 in T CD4+ conv and Foxp3lo cells ↓pSTAT5 in Foxp3hi CD4+ T cells [110] ↓Jak3/STAT5 phosphorylation in naive CD4+ T cells [111]
STAT6	↑ STAT6 mRNA in PBMC [26] ↓pSTAT6 in CD8+ T cells [41]	?
CIS	?	↑ CIS mRNA in PBMC in active SLE [114]
SOCS1	↓ SOCS1 [45] ↑ SOCS1 mRNA in PBMC in IFNβ treated patients [47]	↑SOCS1 in T cells [86] SOCS1 mRNA unchanged in PBMC [114] ↓ SOCS1 mRNA in PBMC [112] ↑ SOCS1 mRNA in CD4+ T cells [115]
SOCS3	↑ SOCS3 [46,51]	↑ SOCS3 in CD4+ T cells [104] SOCS3 mRNA unchanged in PBMC [114]
SOCS5	↓ SOCS5 [45]	?
SMAD4	SMAD4 mRNA unchanged in CD4+ T cells [63] ↑SMAD4 mRNA in CD4+ T cells [64]	?
SMAD7	↓ SMAD7 mRNA in CD4+ T cells [63,64]	?
TNFα adapter proteins	↑ FADD mRNA in leukocytes [66] ↑TRAF2 mRNA in leukocytes [67]	↓TRADD, FADD, RIP-1, TRAF-2 mRNA in PBMC [123]
